# Preparation and evaluation of goose reovirus inactivated vaccine

**DOI:** 10.1186/s12917-017-1134-0

**Published:** 2017-07-06

**Authors:** Xiaoyu Niu, Bingqian Zhang, Xianglong Yu, Xin Zhang, Yanguo Dou, Yi Tang, Youxiang Diao

**Affiliations:** College of Animal Science and Technology, Shandong Agricultural University, Daizong Road 61, Tai’an, Shandong 271018 China

**Keywords:** Reovirus, Goose, Inactivated vaccine

## Abstract

**Background:**

Infection with Goose Reovirus (GRV) can cause serious economic losses in the goose breeding industry. In this study, the GRV allantoic fluid was concentrated and used as an antigen in a formalin-inactivated oil-emulsion vaccine.

**Results:**

When 6 day-old geese were inoculated, antibodies against GRV became detectable at 6 days post-vaccination, their concentration peaked at 3 weeks. These antibodies were maintained for longer than 2 weeks. As the most susceptible age for GRV infection is birds under 2 weeks of age this vaccine should provide adequate cover for the most at risk birds. When geese were exposed to reovirus at different time intervals after immunization, the data revealed that the vaccine can provide a protection rate of 80%. The developed vaccine has good stability and could be stored at 4 °C for at least 12 months.

**Conclusion:**

These results indicate that the developed GRV vaccine is safe, effectively absorbed, efficacious in inducing a rapid immune response, and effective in controlling GRV infection. Our results should be useful for the application of vaccines for controlling GRV in different goose flocks.

**Electronic supplementary material:**

The online version of this article (doi:10.1186/s12917-017-1134-0) contains supplementary material, which is available to authorized users.

## Background

Avian reovirus (ARV) belongs to the genus *Orthoreovirus* within the family *Reoviridae* [1, 2]. Avian reovirus was first isolated from the respiratory tract of a chicken with chronic respiratory disease, liver necrosis, and arthritis by Fahey and Crawley in 1954 [3]. In 1972, Walke et al. confirmed the virus as a “reovirus” [4]. Researchers subsequently identified ARV infection in South Africa, France, Israel, Italy, China, and many other countries [5–7]. In 1997, the main Muscovy duck breeding region in China was affected by an outbreak of duck reovirus (DRV) disease, which resulted in white spots on the liver. Then, the duck reovirus was isolated from ducks in other breeding regions. Previous research has shown that the degree of identity at the sigma C protein level between the deduced amino acid sequence of the DRV and ARV was only 21–22% [8] and serological data showed that the DRV was antigenically different from the Avian Reoviruses present in chickens [9, 10]. All these above suggest that the DRV is very different from ARV and should be classified separately.

It has been reported that GRV can cause viral arthritis in geese, and infected geese often exhibited spleen inflammation, while those with subacute or chronic onset showed pericarditis, arthritis, and/or tenosynovitis [7]. The GRV induced clinical symptoms appeared at 2–3 weeks of age, and the disease course lasted 3–6 weeks [11]. GRV has been isolated from various organs/tissues of infected geese [7]. A previous experiment showed that the virus strains from ducks and geese shared high homology in the S1 genome segment and [12] physico-chemical, clinical and biological characteristics and molecular properties were also identical to each other [7]. Based on sequence data currently available and on criteria established for demarcation of reovirus species, previous reports [7] believe that GRVs and DRVs should be classified into a common species.

ARVs do not have a capsule membrane; instead, they have a double-capsid structure and spherical symmetry of 20 bodies. The virus particle diameter is approximately 60–80 nm. Its buoyant density in cesium chloride (CsCl) is 1.36 g/mL [13–15]. It does not aggregate red blood cells of the chicken, turkey, duck, goose, rat, or mouse [12, 16]. The virus also has the capacity to survive at low temperatures [17]. The viral genome is linear double-stranded RNA, which is composed of 10 genome segments. The genome segments were divided into three groups according to the different electrophoretic mobility of the fragments. There are three large gene segments (L1, L2, L3), three medium-sized gene segments (M1, M2, M3), and four small gene segments (S1, S2, S3, S4) [18–21]. The virus recombination rate is as high as 3%–5% [22].

GRV can replicate upon inoculation into goose embryos in the yolk sack, allantoic cavity, and allantoic membrane, the most effective option of which is inoculation in the yolk sac. Embryos inoculated with the virus generally die within 7 days [23–25]. The dead embryos show systemic bleeding, allantoic membrane thickening, and clear allantoic fluid [26, 27]. Those embryos that do survive more than seven days exhibit necrosis of the liver and spleen. Reovirus can cause significant inhibition of growth in birds, the age group of birds between one day and 9 weeks of age [28]. Reovirus transmission can be classified into two types: horizontal and vertical. The presence of the virus in the cloaca of injected hens has been confirmed, but the rate of vertical transmission in this context is low [29].

GRV disease has become common in many provinces of China. The reovirus can also occur as a co-infection with other pathogenic microorganisms, making the GRV infection more serious [20]. The most effective way to prevent the disease is vaccination. However, there are no vaccines available for GRV prevention. To bridge this gap, in this study, an oil emulsion inactivated vaccine for GRV was developed. The antibody responses in immunized geese were detected by an indirect ELISA method that was established in our laboratory. The results of challenge experiments demonstrated the efficacy of the inactivated GRV vaccine. The success of this study lays the foundations for the prevention and control of the disease, which should have major significance for the vitality of the goose industry.

## Methods

### Virus and animals

The SPF chicken embryos were purchased from the Poultry Research Institute of Shandong Academy of Agricultural Sciences. The geese and goose eggs were purchased from a company in Jinan, Shandong. The geese used in the study were allowed to acclimatize to their new surroundings and were healthy at the start of the experiment. Serum samples were collected from geese prior to challenge or immunization to confirm that these animals were free of GRV antibody via the ELISA assay.

Goose reovirus was isolated at our laboratory from the liver and spleen of infected geese from Jiangsu province in China, after several passages via the chorioallantoic membrane (CAM) of GRV antibody-negative goose eggs. The strain was named JS-01, the sigma C gene sequence of which has been deposited with the Genbank accession number MF139036. The virus was titrated on fertile SPF chicken eggs. The GRV liquid was subjected to a series of tenfold dilutions from 10^−1^ to 10^−5^, and each dilution was inoculated in 9-day-old SPF chick embryos. The egg lethal dose 50 (ELD50) was calculated by the method of Reed and Muench (1938).

### Pathogenicity of the JS-01 strain

The experimental geese were housed in SPF animal isolators that were ventilated under negative pressure, and were provided with feed and water ad libitum. Inoculations of 6 day-old geese were performed intramuscularly with 0.2 ml JS-01 strain, which contained 10^–3.75^ ELD50. 20 geese were infected to investigate the pathogenicity of JS-01, and another 15 birds were left uninoculated as controls. Goslings in each group were inspected daily for signs of disease. Three birds per group were killed and necropsied on days 4, 6, 8,10 and 14 post injection.

### Preparation of oil-adjuvant inactivated GRV vaccine

The GRV JS-01 strain was propagated via the CAM of GRV antibody-negative goose eggs. Eggs that died within 24 h were abandoned. Allantoic fluid of the remaining eggs was collected at 120 h post inoculation as the virus fluid. The allantoic fluid was inactivated by adding 0.2% formalin (*v*/v) and kept at 37 °C for 36 h, shaken every 8 h. The formalin-treated virus fluid was inoculated into SPF chicken embryos, and the harvested allantoic fluid, after three passages, was used to perform reverse transcriptase PCR (RT-PCR) for GRV detection to determine whether the virus was inactivated totally or not.

Firstly, 184 ml sterilized mineral, 12 ml span-80 (Sorbitan (Z)-mono-9-octadecenoate, ThermoFisher, USA) and 4 ml stearic acid aluminum were mixed uniformly as the oil phase. Then the oil phase and 200 ml inactivated virus fluid, which contains sterilized 4% tween-80 (Polysorbate-80) were churned at 3000 round per minute (rpm) for 30 min with homogenizer (voshin, Jiangsu China).

### Purity of vaccine

Tests for microbial contaminants included mycoplasma examination and virus RT-PCR. The vaccine was innoculated onto Thiolglycollate medium (T.G) and Soybean-Casein Digest Agar Medium (TSA) cultivated at 37 °C and examined for microbial colony formation.

The vaccine was subcultured in modified Frey’s medium to perform the mycoplasma testing [30, 31].

RT-PCR assay was carried out to detect the presence of Avian Influenza virus and Newcastle disease Virus (Table 1).

**Table 1:** Primes used in the RT-PCR assay to detect the presence of virus

Virus		Sequence
GRV	GRV-F	TGAGACGCCTGACTACGATT
	GRV-R	ATGCTTGGAGTGAGACGACT
AIV	AIV-F	GGAGGTTGGTCAGGATTAGTTG
	AIV-R	ACAAGAGATGAGGCGACAGT
NDV	NDV-F	AGGGACTGAAGAGGAGGATT
	NDV-R	TGAGTGTGATTGTATTAGGTGG

### Immunization and challenge procedures

Ninety geese were randomly divided into three groups (A, B and C) and kept in SPF animal isolators that were ventilated under negative pressure with consistent conditions (Table 2). Geese from group A and B were injected intramuscularly with GRV vaccine preparations at a volume of 0.2 (5.9276 × 10^−5^ELD_50_) or 0.5 (1.4819 × 10^−4^ELD_50_) mL, while group C were injected with 0.5 mL of PBS via the same route as challenge group. All geese were challenged intramuscularly with 0.2 ml of the JS-01 strain, which contained 10^–3.75^ELD_50_ at 7, 12, and 27 days after vaccination, five geese each time. After challenge, the birds were kept separately. Cloacal and oropharynx swabs were taken from each group on days 3, 7, and 12 days post injection, and viral shedding was tested by virus isolation and the RT-PCR assay. All geese including those died during the observation period were weighed twice a week and examined for gross lesions.

**Table 2:** 90 birds divided into three groups (A, B and C) were used to test protective efficacy of vaccine. A4, B4 and C4 were kept to collect serum for antibody detecting without challenging. All groups were kept in different SPF animal isolator

Group		Immunisation dose (intramuscularly)	Challenge at (days post immunisation)
A	A1	0.2 ml vaccine	7
	A2	0.2 ml vaccine	12
	A3	0.2 ml vaccine	27
	A4	0.2 ml vaccine	none
B	B1	0.5 ml vaccine	7
	B2	0.5 ml vaccine	12
	B3	0.5 ml vaccine	27
	B4	0.5 ml vaccine	none
C	C1	0.5 ml PBS	7
	C2	0.5 ml PBS	12
	C3	0.5 ml PBS	27
	C4	0.5 ml PBS	none

### Sample collection, RNA extraction and nucleotide sequencing

Samples of liver and spleen were collected from the dead animals in each group and processed for the microscopic examination of lesions. Oropharyngeal and cloacal swabs were collected on days 3, 7, and 12 post-challenge (p.c.) for virus isolation and testing for viral shedding, and geese were observed for disease signs and death for 2 weeks p.c.. The swabs were immediately placed in 0.5 mL PBS (pH 7.2) and extensively vortexed. The swab fluids were freeze thawed three times, sterilized by passage through a 0.22 mm filter and stored at −20 °C for further virus isolation via injecting goose embryos. Viral RNA was extracted from the allantoic fluid harvested from the goose embryos used for virus isolation or homogenate using the Tranzol RNA Extraction Kit (TransGen Biotech, Beijing, China) according to the manufacturer’s instructions. The first-strand cDNA was generated from extracted RNA solution extracted using the Reverse Transcriptase M-MLV (Takara, Dalian, China) with Random Primer (pd(N)6, Takara, Dalian, China). The PCR mix containing 0.25 μL EX Taq polymerase (5 U/mL), 5 μl of 10× EX Taq Buffer (Mg^2+^ Plus), 4 μL of dNTP mixture (2.5 mM each dNTP) and 1 μL of each of the two primers (20 μM) was brought to 50 μL with RNase-free water. The reaction conditions were as follows: initial denaturation at 94 °C for 5 min, followed by 35 cycles of denaturation at 94 °C for 1 min, annealing at 54 °C for 30 s and extension at 72 °C for 2 min, with a final extension for 7 min at 72 °C. The RT-PCR products were purified, cloned into pMD18-T vector (TaKaRa, Dalian, China) and sequenced by BGI (Shenzhen, PR China).

A separate set of samples from tissues, such as liver and spleen were fixed in 10% neutral buffered formalin and embedded in paraffin wax, then cut into 4 mm thick sections. Sections were stained with hematoxylin and eosin for microscopic examination.

Before the challenge, sera were obtained from 10 geese from the control and vaccinated groups. After the challenge, five serum samples were also collected from the three groups. The serum samples were collected every three days in each week and evaluated for the development of an immune response against GRV using indirect ELISA. All serum samples were stored at −20 °C.

Alignment of JS-01 strain oC gene with selected reference sequences was performed using Clustal W method of MegAlign program of DNAStar software (version 7.10). A phylogenetic tree was assembled using the neighbor-joining method of the MEGA program (version 5.0) with absolute distances following 1000 bootstrap replicates [32].

### The procedure for detecting GRV antibody by ELISA

A total of 100 μL of GRV fluid, harvested from goose eggs and concentrated by PEG20000, in 0.05 M carbonate buffer (pH 9.6) was coated onto the wells of an ELISA plate (Greiner Bio-One, Germany), which was incubated at 37 °C for 2 h. These plates were blocked by phosphate-buffered saline (PBS) containing 0.5% Polyoxyethy-lene(20)Sorbaitan Monolaurate (Tween-20, ThermoFisher, USA) and 5% powdered milk (solarbio, Beijing, China, *w*/*v*) and incubated at 37 °C for 1 h. After blocking, diluted goose serum (1: 10) was added into wells (200 μl/well) and incubated at 37 °C for 1 h. Afterward, the plates were washed three times with PBS and incubated with rabbit anti-goose IgG (1: 800) conjugated to horseradish peroxidase (HRP, Sigma-Aldrich) at 100 μl/well. The plates were, again, incubated at 37 °C for 1 h, washed three times with PBS, and 3′, 3′, 5′, 5′-tetramethylbenzidine substrate solution (TransGen, Beijing, China) was added into each well (100 μl/well). 50 μl of 3 M H_2_SO_4_ (Wuxi JINGKE Chemical CO., LTD.) was added to stop the reaction, and the optical density (OD) values were observed at 450 nm using an automated ELISA plate reader (Thermo, Waltham, MA, USA). The cut off value of the ELISA assay was an OD of 0.37 at 450 nm.

The rabbit anti-goose IgG was prepared by our lab. In order to isolate IgG from the goose egg yolk, we used the method of chloroform extraction, salt precipitation and DEAE chromatography. Sodium sulphate polyacrylamide gel electrophoresis indicates that we derived relative pure IgG. Rabbits were inoculated with the purified IgG 4 times in all. Serum from the rabbits was collected and purified IgG precipitated with saturated ammonium sulphate. Finally, the purified rabbit IgG was labeled with HRP.

### Statistical evaluation

The data were analyzed by GraphPad Prism version 5.0 software (GraphPad Software Inc., San Diego, CA). To investigate the differences of the antibody titre among three groups, the data was analyzed using ANOVA via the method of Bonferroni. The *p* values <0.025 were considered to be statistically significant (SPSS Statistics 17.0, IBM, USA).

## Results

### Alignment and phylogenetic tree

**Fig. 1:**
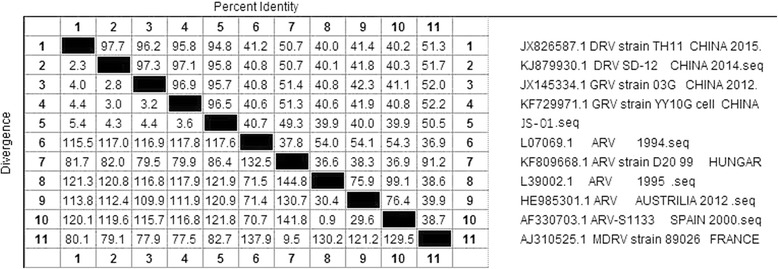
Alignment of the σC gene sequence among JS-01 and other avian reovirus strain

**Fig. 2:**
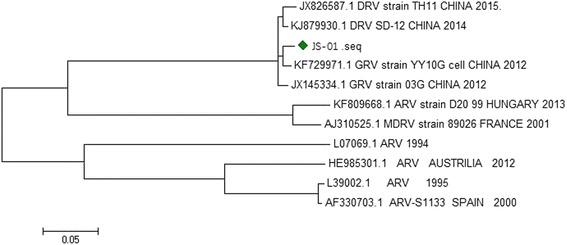
Phylogenetic tree constructed using the neighbor-joining method, based on the sequence of the σC gene of virus strains isolated in the study and other waterfowl reovirus isolates available in GenBank database

The virus strain used in the study was sequenced for the σC gene, which shared homology of 94.8% ~ 96.5% with the waterfowl reovirus (Fig. 1). The σC gene of JS-01 (GenBank accession: MF139036) isolated in the study has been clustered, and it9 was in the same clade with other goose reovirus strains isolated from China. Among them, the YY10G strain (Genebank accession: KF729971.1) isolated in China shared the closest relationship with JS-01 strain (Fig. 2).

### Pathogenicity of the JS-01 strain

The experimental infection of goslings resulted in mortality of 85% (17/20). Acute deaths occurred at the 2nd and 3rd day post injection. Those birds still alive showed general malaise and exhibited lameness during the whole period of observation. Splenomegaly and necrotic foci in the liver and spleen were obvious on post mortem. Virus was detected from the inoculated birds, dead and alive, from homogenate of liver and spleen via RT-PCR assay.

### Response to challenge

**Fig. 3:**
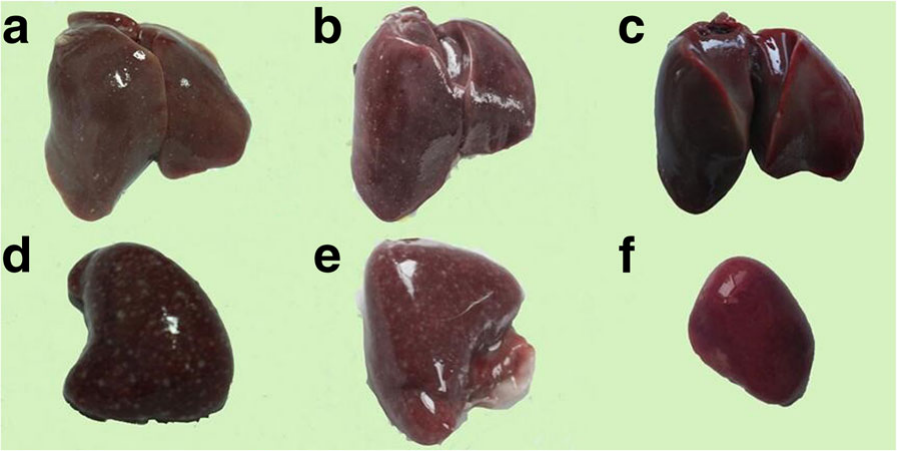
Necropsy of geese infected with JS-01 strain. **a** and **b** Liver hemorrhage and the appearance of white necrotic spots. **d** and **e** Spleen sclerosis, with a large number of necrotic spots appearing in the spleen. **c** liver of healthy a goose from vaccinated group. **f**: spleen of a healthy goose from vaccinated group

**Fig. 4:**
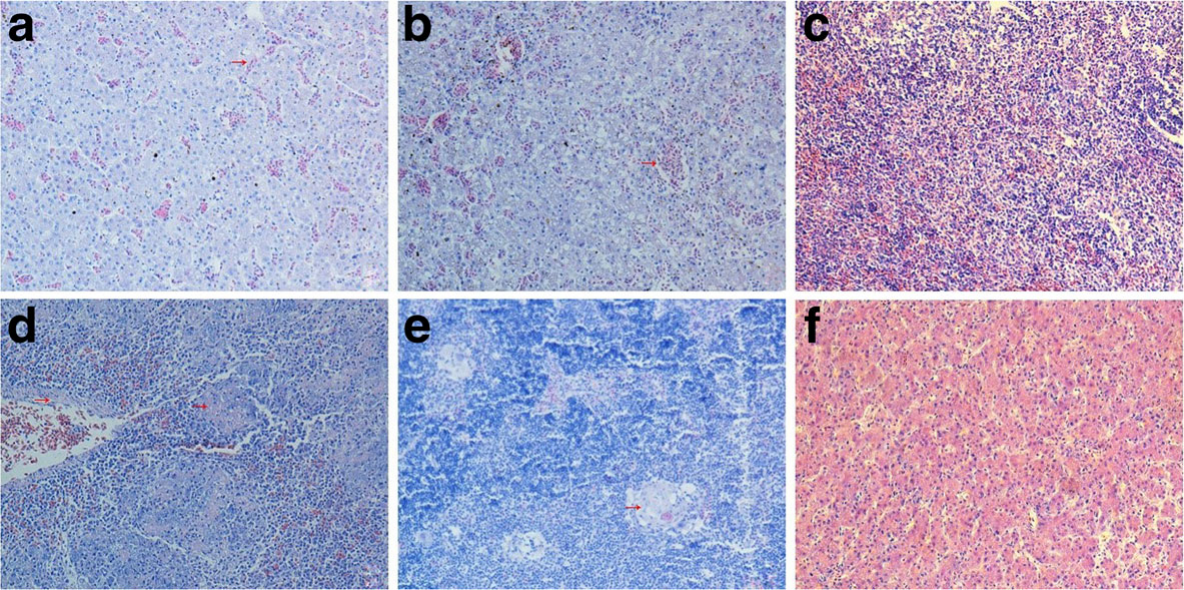
The histopathological changes of geese infected with JS-01 strain. **a** Punctate hemorrhage of liver cells, atrophy of hepatic acini, and necrosis. Inflammatory cell infiltration. **b** Necrosis of liver cells and vacuolization. **c** Intact structures of hepatic lobules, no degeneration and infiltration of lymphocytes. **d** Spleen cell necrosis and cellular hyperplasia. **e** Spleen cell hemorrhage. **f** Arranged densely lymphocytes, no necrosis

To investigate the protective efficacy of the vaccine developed in the study, geese were challenged at different time points post vaccination. The results of these experiments are summarized in Table 3. At post-mortem examination, geese that died in the period of observation showed swelling of the kidneys, liver and spleen; necrotic foci were present in the last two organs (Fig. 3 and Fig. 4).

**Table 3:** Description of waterfowl parvovirus isolates involved in this study

Strain	GenBank accession	Host	Separatum	Year
JS-01	MF139036	Goose	China	2016
TH11	JX826587.1	Duck	China	2015
SD-12	KJ879930.1	Duck	China	2014
03G	JX145334.1	Goose	China	2012
YY10G	KF729971.1	Goose	China	2012
Somerville 4	L07069.1	Chicken	Australia	1994
D20/99	KF809668.1	Chicken	Hungary	2013
S1133	L39002.1	Chicken	Australia	1994
12–1167	HE985301.1	Chicken	Australia	2012
S1133	AF330703.1	Chicken	Spain	2000
89,026	AJ310525.1	Muscovy duck	France	2001

### Antibody response of vaccinated geese

**Fig. 5:**
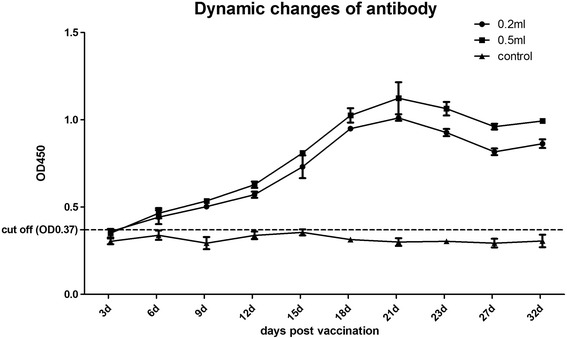
The dynamic changes in antibodies induced by the inactivated vaccine in geese. One-week-old geese were injected intramuscularly (i.m.) with 0.2 or 0.5 mL of formalin-inactivated vaccine, and sera were collected randomly from six geese every three days for GRV antibody detection. The bars indicate the standard deviation

Before the vaccination, GRV antibody was measured by the ELISA assay. The antibody level began to rise markedly at 6 d.p.v., and peaked after 3 weeks (Fig. 3). During the next two weeks, the ELISA values fell slowly, but still remained in the positive range and were much higher than the cut-off value (Fig. 5). The age of the highest susceptibility to GRV infection is from 1 to 4 weeks old, therefore the results demonstrate that the vaccine can provide protection rate over 80% in susceptible birds.

The results showed that, the group immunized with 0.5 ml of vaccine acquired protection rate of 80% against GRV (Table 4). Taking the titer of antibody into consideration, (Table 4), when the ELISA values of serum antibody increased to 0.60 or higher, the geese were completely protected. These results also demonstrate that geese were protected from the viral challenge for at least 1 month after inoculation, which is sufficient to cover the whole of the highly infectious period.

**Table 4:** Results of viral shedding after the challenge Results showed that birds vaccinated with 0.5 ml of vaccine at 12 dpi can be completely protected from GRV. While vaccinating with 0.2 ml and 0.5 ml of vaccine can also provide complete protection

Challenge time (dpv)	Group	Virus isolation from the swabs collected on different days after challenge: positive/total						Survival rate	Protection rate	OD value
		Day 3		Day 7		Day 12				
		Oropharyngeal	Cloacal	Oropharyngeal	Cloacal	Oropharyngeal	Cloacal			
7	0.2 mL	1/5	1/5	0/4^a^	0/4^a^	0/4^a^	1/4^a^	80%	60%	OD 0.45
	0.5 mL	0/5	0/5	0/5	0/5	0/5	1/5	100%	80%	OD 0.47
	Control	0/5	5/5	0/3^a^	3/3^a^	0/1^a^	1/1^a^	20%	0%	OD 0.34
12	0.2 mL	0/5	1/5	0/4^a^	0/4^a^	0/4^a^	0/4^a^	80%	80%	OD 0.57
	0.5 mL	0/5	0/5	0/5	0/5	0/5	0/5	100%	100%	OD 0.60
	Control	0/5	5/5	0/2^a^	1/2 ^a^	0/2^a^	1/2 ^a^	40%	20%	OD 0.29
27	0.2 mL	0/5	0/5	0/5	0/5	0/5	0/5	100%	100%	OD 0.82
	0.5 mL	0/5	0/5	0/5	0/5	0/5	0/5	100%	100%	OD 0.96
	Control	0/5	4/5	0/3^a^	0/3 ^a^	0/3^a^	0/3 ^a^	60%	60%	OD 0.30

^a^ The sample number was less than 5 because geese had died. Complete protection: no viral shedding and death in the group; partial protection: viral shedding or/and death in the group

### Viral shedding and virus isolation after challenge

To test for viral shedding, oropharyngeal and cloacal swabs were collected for virus isolation via goose embryos. Harvested allantoic fluid was detected by RT-PCR. Positive results in the RT-PCR indicated that the geese were shedding virus. (dates are shown in Table 4).

## Discussion

In this experiment, we developed a GRV inactivated vaccine and evaluated its safety and efficacy in protecting goslings from GRV infection. The ELISA results showed that the antibody level in innoculated birds increased to OD0.6 or higher, and the vaccine exhibited a strong protective effect in geese.

When inoculated with 0.5 ml vaccine at 7 dpi, it can provide 80% protection. However, innoculation at 12 dpi with 0.5 ml vaccine can providing 100% protection. (Table 4).

Inactivated vaccines are used to control many infectious diseases in the poultry, due to their readily availability. In addition, inactivated whole-virus vaccines has been confirmed to be safe and high efficacy [33, 34]. However there has been little research into the protective immune response to GRV vaccines, possibly as the disease is newly emergent and largely present in China.

Compared with the live virus vaccines, the inactivated vaccine preparation does not appear to be toxic to goslings and produces a protective antibody response in inoculated birds for a length of time that would protect from natural disease. Selection of the inactivation agent is the most important factor in the production of an inactivated vaccine. Ideally, inactivation involves the virus completely losing its activity while maintaining good immunogenicity; therefore, there is a strong relationship between the effect of inactivation and the choice of the inactivated agent. Formaldehyde is the most commonly used inactivation agent for RNA viruses. Considering that ARV is resistant to low temperatures and a low concentration of formaldehyde solution, in this study, we chose reaction conditions of 0.2% formaldehyde and incubation at 37 °C.

**Fig. 6:**
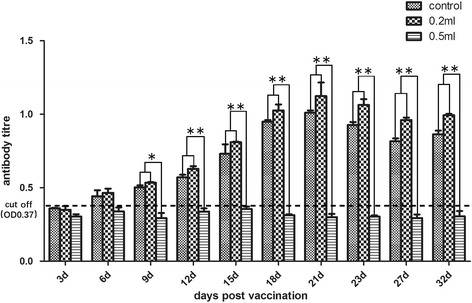
Statistical significance of antibody titre between vaccinated group and control group. The data was analyzed via ANOVA (method of Bonferroni). * *p* < 0.025; ** *p* < 0.001

An indirect ELISA was developed using concentrated virus as the antigen. Serological responses following vaccination, as measured by this ELISA, were effective in most birds and correlate closely with protection. Over the whole observation after vaccination, the antibody titre of the control group against GRV was significantly lower when compared with vaccinated groups (*p* value <0.025, Fig. 6).

Previous study has confirmed the efficacy of oil-emulsion inactivated vaccines for protecting ducks from DRV [35]. Efficacy evaluations have been based on a challenge study performed a few weeks after vaccination, when the antibody titers reach or are around their peak. Our study has indicated that the vaccinated geese could be completely protected from GRV challenge when the antibody titers to the challenge virus equaled or were greater than OD0.6, a titer that can be easily induced by inactivated vaccine immunization.

Horizontal transmission is a major route of infection for GRV. The elimination time of GRV from the intestinal and respiratory tracts is long, especially in the intestines, which means that fecal pollution is a major source of infection [36, 37]. Specifically, infection can quickly spread through feces in the same house. In this study, young geese immunized by the inactivated vaccine showed a high protection rate, which is over 80%, against viral challenging. Thus, we believe that the vaccine can aid in preventing the disease from spreading.

The data indicate that the inactivated GRV vaccine may represent an effective method for controlling GRV infection in the goose industry. However, vertical transmission is also an important route of GRV infection. Vertically transmitted GRV causes great economic losses due to mortality of the affected offspring, although infected breeding geese do not show clinical signs. It could thus have significant economic benefits for the industry if immunizing laying breeders could prevent vertical transmission [38]. In chickens the immune effect is generally better for breeding hens if inoculation with a living vaccine is performed before that with an inactivated vaccine [29]. We plan to study the immune effect of this vaccine in breeding geese in future work.

In this context, it has yet to be studied whether our vaccine would interfere with other vaccines for goose diseases, but this is worthy of investigation in future studies. We also need to develop and improve immunization programs for geese field use. Nonetheless, this study has provided a useful foundation by showing that the inactivated GRV vaccine could control and prevent infection.

## Conclusion

The present work demonstrated that immunization with the new inactivated vaccine provides protective immunity to goslings, thereby reducing mortality, morbidity, and fecal shedding through challenge-exposure with virulent GRV. Since the advent of new, effective, and safe GRV vaccines on the market is in urgent in the near future to combat GRV. We should bear in mind that full biosecurity and husbandry management in the field can maximize vaccine effectiveness for prevention and control of GRV.

## Additional file

Additional file 1: Alignment data of the sequence among JS-01 strain and other reovirus strains. (TXT 61 kb)

## Abbreviations

ANOVA: Analysis of variance
ARV: Avian reovirus
CAM: Chorioallantoic membrane
DRV: Duck reovirus
ELD50: Egg lethal dose 50
ELISA: Enzyme linked immunosorbent assay
GRV: Goose reovirus
HRP: Horseradish peroxidase
OD: Optical density
p.c.: Post-challenge
PBS: Phosphate buffered saline
PCR: Polymerase chain reaction
PEG: Polyethylene glyco
RNA: Ribonucleic acid
RT-PCR: Reverse transcriptase PCR
SPF: Specific pathogen free
T.G: Thiolglycollate medium
TSA: Soybean-Casein Digest Agar Medium
